# Best practice in major elective rectal/pelvic surgery: enhanced recovery after surgery (ERAS)

**DOI:** 10.1007/s13304-017-0492-2

**Published:** 2017-10-24

**Authors:** Josefin Segelman, Jonas Nygren

**Affiliations:** 1 Department of Molecular Medicine and Surgery, Karolinska Institutet, Stockholm, Sweden; 2 Department of Surgery, Ersta Hospital, Box 4622, 116 91 Stockholm, Sweden; 3 Department of Clinical Sciences, Danderyd Hospital, Karolinska Institutet, Stockholm, Sweden

**Keywords:** ERAS, Perioperative care, Rectal surgery, Pelvic surgery

## Abstract

Within traditional clinical care, the postoperative recovery after pelvic/rectal surgery has been slow with high morbidity and long hospital stay. The enhanced recovery after surgery program is a multimodal approach to perioperative care designed to accelerate recovery and safely reduce hospital stay. This review will briefly summarize optimal perioperative care, before, during and after surgery in this group of patients and issues related to implementation and audit.

## Introduction

Major rectal surgery, involving rectal resections below the peritoneal reflection (i.e., 8–9 cm anteriorly in females and 9–10 cm in men) [1], have been associated with significant morbidity, prolonged hospital stay and unplanned readmissions. To reduce surgical stress and facilitate postoperative recovery, enhanced recovery after surgery (ERAS) principles have been introduced [2]. ERAS is a dynamic protocol including a series of evidence-based treatments covering the entire perioperative period. The implementation of ERAS-care pathways improves the quality of surgical care and accelerates the recovery, with strong evidence for patients undergoing open colonic resection [3]. The more demanding and complex rectal resections, however, involve unique complications, higher complication rates and extended length of hospital stay compared with colonic resections. Several (but not all) of the ERAS interventions recommended for colonic surgery, have been successfully applied to rectal resections [4]. The ERAS guidelines for this population are summarized in this review.

## Methods

This is not a meta-analysis or a systematic review, but an updated review of available evidence for ERAS perioperative care in rectal surgery. Papers indexed using the MESH terms ERAS, enhanced recovery, colorectal, rectal, surgery were reviewed and relevant papers has been reported in this review, especially in relation to the International ERAS guidelines in elective rectal/pelvic surgery published in Clinical Nutrition 2012 [5].

## ERAS: preparing the patients before surgery

Preadmission information, education and counseling about surgical and anesthetic procedures reduce fear and anxiety and enhance postoperative recovery and discharge [4]. The information includes the role of perioperative feeding, early postoperative mobilization, pain control, and respiratory physiotherapy to reduce the prevalence of complications. A large proportion of patients undergoing rectal surgery will receive a diverting or permanent stoma. To better prepare for the procedure and to reduce length of stay in hospital, patients should have preadmission education regarding enterostomal therapy by a stomal therapy nurse. Marking of the stomal site should be made preoperatively.

Outcomes are improved by preoperative identification of risk factors and evaluation and optimization of comorbidity, such as anemia, diabetes mellitus and hypertension [4]. Malnourished patients benefit from preoperative nutritional supplementation with fewer infectious complications and anastomotic leaks [6, 7].

Patients should stop smoking or drinking excessive amounts of alcohol [4].

Mechanical bowel preparation (MBP), such as oral sodium phosphate, is stressful for the patient and can result in dehydration and changes in electrolyte balance. In colonic surgery, it is associated with prolonged postoperative ileus. A Cochrane review show that MBP has no benefit in colorectal surgery in terms of leakage of the bowel anastomosis, and it was concluded that routine bowel preparation should be avoided [8]. However, the situation might be different in patients receiving a diverting stoma when undergoing resection of the rectum with restoration of bowel continuity. For this group of patients, a recent randomized controlled trial reported a trend towards a higher risk of anastomotic leak and peritonitis in patients randomized to no MBP compared with the MBP group [9]. Until further studied, MBP is recommended in patients undergoing rectal resection with diverting stoma. In pelvic surgery in general, MBP should not be used.

## ERAS: the day of surgery

Fasting after midnight has been standard in elective surgery. The purpose has been to avoid pulmonary aspiration of gastric contents during general anesthesia. However, no scientific evidence supports this practice, and there is today robust evidence that intake of clear fluids up to 2 h before surgery does not increase the prevalence of complications [10]. Intake of clear fluids up to 2 h and solid food up to 6 h prior to induction of anesthesia is recommended.

### Preparation in the morning before surgery

Provision of a clear fluid containing a defined concentration of complex carbohydrates 2 h before anesthesia decreases preoperative thirst, hunger and anxiety. Postoperative insulin resistance and catabolism are reduced and clinical outcome is improved, including reduced hospital stay [11]. Preoperative oral carbohydrate loading should be administered unless patients demonstrate increased risk of aspiration, such as bowel obstruction. The benefits in patients with diabetes mellitus need to be studied in more detail.

Patients should not routinely receive long- or short-acting sedative medication before surgery as it delays immediate postoperative recovery. Short-acting benzodiazepines can be used in young patients before potentially painful interventions such as insertion of spinal or epidural catheter, but they should not be used in the elderly (age >60 years) [12, 13].

Pharmacological prophylaxis against venous thrombosis reduces the prevalence of symptomatic venous thromboembolism without increasing the risk of bleeding. Patients with extensive comorbidity, malignant disease and who are taking corticosteroids preoperatively are at an increased risk. Patients should wear compression stockings and receive pharmacological prophylaxis with low-molecular-weight heparin. Extended prophylaxis for 28 days should be considered in patients with colorectal cancer or other patients with an increased risk of venous thromboembolism [4].

Prophylactic antibiotics reduce the prevalence of infectious complications in colorectal surgery [14]. Patients should receive a single dose of antimicrobial prophylaxis before skin incision. Repeated doses may be necessary depending on the half-life of the drug and duration of surgery. Skin should be prepared with chlorhexidine–alcohol as this prevents surgical site infection [4].

### Surgery and anesthesia

A more detailed description of the recommended standard anesthetic protocol is given in the ERAS Society recommendations for pelvic/rectal surgery [12, 13]. In summary, to attenuate the surgical stress response, intraoperative maintenance of adequate hemodynamic control, central and peripheral oxygenation, muscle relaxation, depth of anesthesia and appropriate analgesia is strongly recommended. The latter includes a thoracic epidural catheter.

It is important to have a strategy for the management of postoperative nausea and vomiting (PONV), to promote early return of oral food intake. Non-smokers, female patients, patients with a history of motion sickness and patients using opioids are at high-risk for PONV. All patients with ≥2 risk factors should receive anti-emetic prophylactic treatment. If patients develop nausea and vomiting, treatment should be via a multimodal approach [12, 13].

Laparoscopic pelvic surgery has been shown to decrease the inflammatory response to surgery compared with the open approach. Safety and disease-specific outcomes have been shown improved or at least equal for benign disease. For malignant disease, recent meta-analysis indicate that there are no difference in long-term oncological outcome after laparoscopic as compared to open surgery for rectal cancer while short term outcome with regard to complications and recovery is improved after laparoscopic surgery [15].

There is good evidence that routine insertion of nasogastric tubes should be avoided in colorectal surgery since they cause increased fever, atelectasis and pneumonia [16].

Prolonged exposure of the body during surgery and impaired thermoregulation by anesthesia can cause hypothermia. Hypothermic patients are at an increased risk of postoperative complications such as wound infections, cardiac ischemia and bleeding. Patient body temperature should be monitored during and after surgery and hypothermia should be avoided [4].

Avoidance of bowel preparation and free oral intake of clear fluids until 2 h before induction of anesthesia reduce dehydration and electrolyte imbalance. During surgery, fluid balance should be optimized by targeting cardiac output. Fluid excess should be avoided. Vasopressors can be used to treat arterial hypotension [12, 13].

Pelvic drains have not been shown beneficial and should not be routinely used [17]. To avoid urinary tract infections, urinary drainage should be as short as possible, and when prolonged catheterization is necessary (>3 days) suprapubic catheters are preferred to transurethral [4].

## ERAS: optimize postoperative care

Postoperative ileus delays recovery and hospital discharge. The following elements have been shown to reduce postoperative ileus: use of thoracic epidural catheter and avoidance of opioids, balancing fluids and optimizing gut function. The latter is achieved by avoidance of nasogastric intubation, minimizing PONV, early intake of oral food, use of chewing gum [18, 19] and laxatives. Multimodal opioid-sparing analgesia reduces postoperative ileus and enhances return of bowel movements. Thoracic epidural anesthesia is recommended for open rectal surgery for 48–72 h. In laparoscopic surgery, intravenous or epidural lidocaine provides adequate pain control. Paracetamol and non-steroidal anti-inflammatory drugs spare opioid use and opioid related side effects. Wound catheters and transversus abdominis plane (TAP) blocks can be used, although the evidence level is limited [12, 13].

**Fig. 1:**
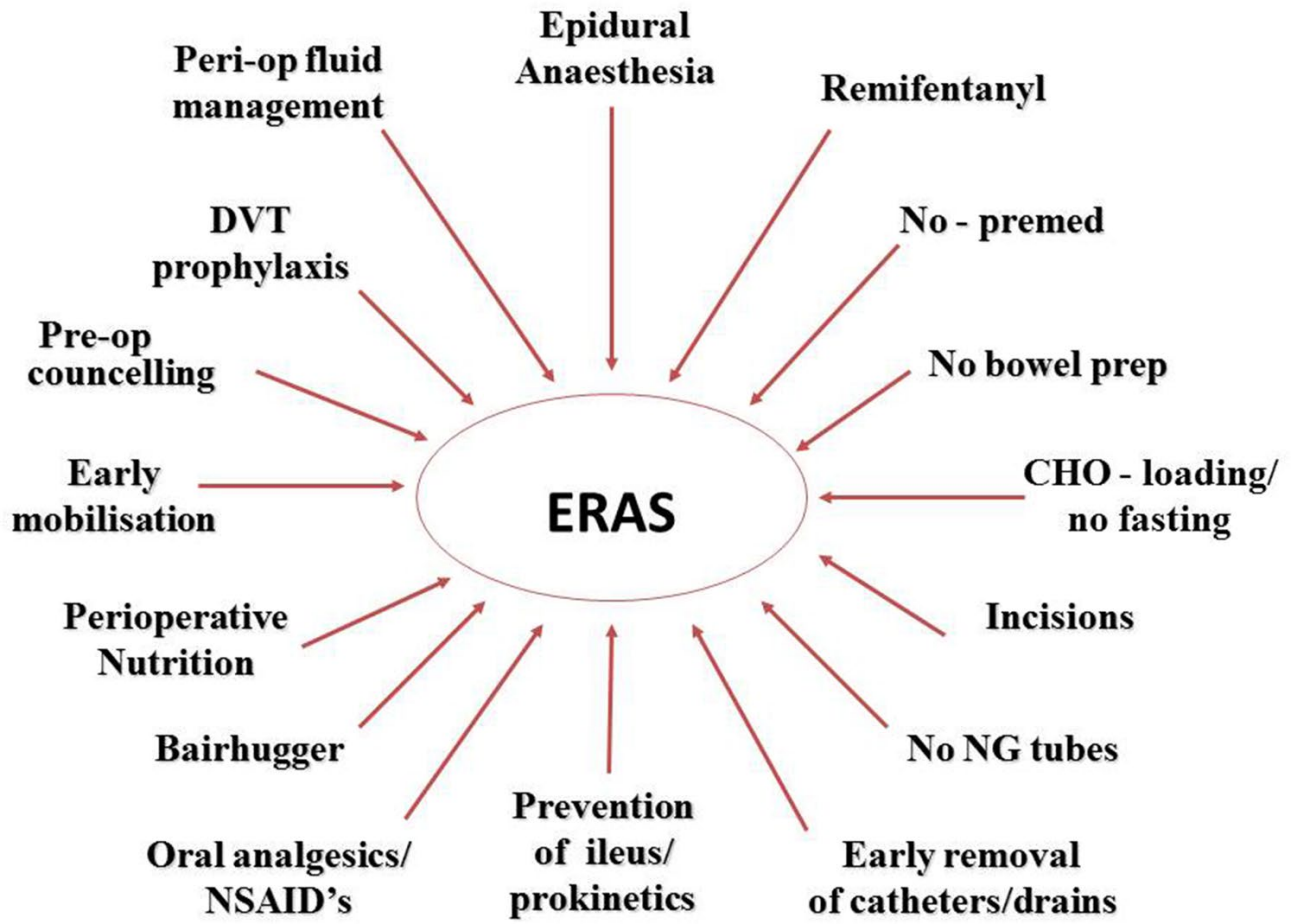
ERAS: multimodal perioperative care protocol

Early oral diet enhances recovery and decreases infectious complications [20]. Postoperative ileus and vomiting should be prevented and free unrestricted oral diet is recommended from 4 h after rectal surgery. Oral nutritional supplements should be offered to maintain sufficient intake of energy and protein. Insulin resistance, a physiological response to surgical trauma, causes postoperative hyperglycemia. Hyperglycemia is harmful with increased morbidity and mortality. Efforts should be made to prevent surgical stress and thereby prevent insulin resistance [6]. Interventions include preoperative oral carbohydrate loading, epidural anesthesia, and minimally invasive surgery [6]. Established hyperglycemia should be actively treated (Fig. 1).

Bed rest is associated with increased risk of thromboembolism, pneumonia, insulin resistance and muscle loss [21]. Early postoperative mobilization decreases pain and ileus. Patients should be nursed in an environment that encourages independence and mobilization. The recommendation is to be out of bed 2 h on the day of surgery and 6 h per day thereafter [4].

## ERAS: implementation, register outcomes and quality control

Issues related to successful implementation of ERAS has been studied repeatedly [22], and a careful audit is vital [23]. Audit and feedback leads to quality improvements in healthcare. Adherence to an established ERAS protocol is proven to be in linear relationship to improved outcomes, including postoperative morbidity as well as 5-year cancer specific survival after colorectal cancer surgery [24, 25]. A systematic audit should measure clinical outcomes (morbidity, length of stay in hospital) and compliance to the protocol. With the multinational efforts of the ERAS Society Research Committee, an audit system has been built to facilitate data collection and implementation of ERAS. This database will serve as an essential background for future modifications of the multimodality concept.

## Outcomes of ERAS

In colorectal surgery, meta-analyses show a positive effect on mortality, morbidity, length of hospital stay and hospital readmissions by application of ERAS principles compared with traditional care [3]. Rectal resections differ, however, from colonic resections. For example chemo radiation for malignancy, immunosuppression for inflammatory bowel disease, creation of a stoma, and flap closures are complicating factors that need to be addressed. In addition, postoperative sequelae such as voiding disturbances, sexual impairment, anal incontinence and the low anterior resection syndrome (LARS) are more common after surgery in the pelvic cavity, and these issues may also be influenced by the use of ERAS interventions such as minimally invasive surgery. There are no randomized trials evaluating the ERAS concept for rectal surgery alone, as published studies include both rectal and colonic surgery. Thus, it is not possible to be definitive about the influence of traditional versus ERAS care in rectal surgery. However, retrospective case-series have suggested a reduction of length of hospital stay by 3–5 days in open [26] and laparoscopic rectal surgery [27]. No increase in complications or mortality has been reported.

## Summary and recommendation

Rectal surgery undertaken within an ERAS program is safe and improves recovery.

## References

[1] Kenig J, Richter P (2013). Definition of the rectum and level of the peritoneal reflection—still a matter of debate?. Wideochir Inne Tech Maloinwazyjne.

[2] Lassen K, Soop M, Nygren J, Cox PB, Hendry PO, Spies C (2009). Consensus review of optimal perioperative care in colorectal surgery: enhanced recovery after surgery (ERAS) group recommendations. Arch Surg.

[3] Varadhan KK, Neal KR, Dejong CH, Fearon KC, Ljungqvist O, Lobo DN (2010). The enhanced recovery after surgery (ERAS) pathway for patients undergoing major elective open colorectal surgery: a meta-analysis of randomized controlled trials. Clin Nutr.

[4] Nygren J, Thacker J, Carli F, Fearon KC, Norderval S, Lobo DN (2013). Guidelines for perioperative care in elective rectal/pelvic surgery: enhanced recovery after surgery (ERAS^®^) society recommendations. World J Surg.

[5] Nygren J, Thacker J, Carli F, Fearon KC, Norderval S, Lobo DN (2012). Guidelines for perioperative care in elective rectal/pelvic surgery: enhanced recovery after surgery (ERAS^®^) society recommendations. Clin Nutr.

[6] Nygren J (2006). The metabolic effects of fasting and surgery. Best Pract Res Clin Anaesthesiol.

[7] Gustafsson UO, Ljungqvist O (2011). Perioperative nutritional management in digestive tract surgery. Curr Opin Clin Nutr Metab Care.

[8] Guenaga KF, Matos D, Wille-Jorgensen P (2011). Mechanical bowel preparation for elective colorectal surgery. Cochrane Database Syst Rev.

[9] Bretagnol F, Panis Y, Rullier E, Rouanet P, Berdah S, Dousset B (2010). Rectal cancer surgery with or without bowel preparation: the French GRECCAR III multicenter single-blinded randomized trial. Ann Surg.

[10] Brady M, Kinn S, Stuart P, Ness V (2003) Preoperative fasting for adults to prevent perioperative complications. Cochrane Database Syst Rev (4):CD004423. doi:10.1002/14651858.CD00442310.1002/14651858.CD00442314584013

[11] Smith MD, McCall J, Plank L, Herbison GP, Soop M, Nygren J (2014). Preoperative carbohydrate treatment for enhancing recovery after elective surgery. Cochrane Database Syst Rev.

[12] Feldheiser A, Aziz O, Baldini G, Cox BP, Fearon KC, Feldman LS et al (2015) Enhanced recovery after surgery (ERAS) for gastrointestinal surgery, part 2: consensus statement for anaesthesia practice. Acta Anaesthesiol Scand 60:289–334 10.1111/aas.12651 PMC5061107 26514824

[13] Scott MJ, Baldini G, Fearon KC, Feldheiser A, Feldman LS, Gan TJ (2015). Enhanced recovery after surgery (ERAS) for gastrointestinal surgery, part 1: pathophysiological considerations. Acta Anaesthesiol Scand.

[14] Nelson RL, Glenny AM, Song F (2009) Antimicrobial prophylaxis for colorectal surgery. Cochrane Database Syst Rev 1:CD001181. doi:10.1002/14651858.CD001181.pub3 10.1002/14651858.CD001181.pub3 19160191

[15] Chen K, Cao G, Chen B, Wang M, Xu X, Cai W (2017). Laparoscopic versus open surgery for rectal cancer: a meta-analysis of classic randomized controlled trials and high-quality nonrandomized studies in the last 5 years. Int J Surg.

[16] Nelson R, Edwards S, Tse B (2004) Prophylactic nasogastric decompression after abdominal surgery. Cochrane Database Syst Rev 3:CD004929. doi10.1002/14651858.CD004929.pub2

[17] Denost Q, Rouanet P, Faucheron JL, Panis Y, Meunier B, Cotte E et al (2017) To drain or not to drain infraperitoneal anastomosis after rectal excision for cancer: the GRECCAR 5 randomized trial. Ann Surg 265:474–480 10.1097/SLA.0000000000001991 27631776

[18] Pereira Gomes Morais E, Riera R, Porfirio GJ, Macedo CR, Sarmento Vasconcelos V, de Souza Pedrosa A (2016). Chewing gum for enhancing early recovery of bowel function after caesarean section. Cochrane Database Syst Rev.

[19] Short V, Herbert G, Perry R, Atkinson C, Ness AR, Penfold C et al (2015) Chewing gum for postoperative recovery of gastrointestinal function. Cochrane Database Syst Rev (2):CD006506. doi:10.1002/14651858.CD006506.pub3 10.1002/14651858.CD006506.pub3 PMC9913126 25914904

[20] Andersen HK, Lewis SJ, Thomas S (2004) Early enteral nutrition within 24 h of colorectal surgery versus later commencement of feeding for postoperative complications. Cochrane Database Syst Rev (4):CD004080. doi:10.1002/14651858.CD004080.pub2 10.1002/14651858.CD004080.pub2 17054196

[21] Kehlet H, Wilmore DW (2002). Multimodal strategies to improve surgical outcome. Am J Surg.

[22] Gramlich LM, Sheppard CE, Wasylak T, Gilmour LE, Ljungqvist O, Basualdo-Hammond C (2017). Implementation of enhanced recovery after surgery: a strategy to transform surgical care across a health system. Implement Sci IS.

[23] Segelman J, Nygren J (2014). Evidence or eminence in abdominal surgery: recent improvements in perioperative care. World J Gastroenterol.

[24] Gustafsson UO, Hausel J, Thorell A, Ljungqvist O, Soop M, Nygren J (2011). Adherence to the enhanced recovery after surgery protocol and outcomes after colorectal cancer surgery. Arch Surg.

[25] Gustafsson UO, Oppelstrup H, Thorell A, Nygren J, Ljungqvist O (2016) Adherence to the ERAS protocol is associated with 5-year survival after colorectal cancer surgery: a retrospective cohort study. World J Surg 40:1741–1747 10.1007/s00268-016-3460-y 26913728

[26] Nygren J, Soop M, Thorell A, Hausel J, Ljungqvist O (2009). An enhanced-recovery protocol improves outcome after colorectal resection already during the first year: a single-center experience in 168 consecutive patients. Dis Colon Rectum.

[27] Branagan G, Richardson L, Shetty A, Chave HS (2010). An enhanced recovery programme reduces length of stay after rectal surgery. Int J Colorectal Dis.

